# Evaluating the dissemination of evolutionary biology concepts in medicine

**DOI:** 10.1590/1414-431X2023e13052

**Published:** 2023-10-27

**Authors:** C. Mermelstein, M.L. Costa, C.C. Coutinho

**Affiliations:** 1 Laboratório de Diferenciação Muscular, Instituto de Ciências Biomédicas, Universidade Federal do Rio de Janeiro, Rio de Janeiro, RJ, Brasil

**Keywords:** Evolution, Medicine, Bibliometrics, Education, Curriculum, Medical

## Abstract

Darwin's theory of evolution, which is based on variation, heredity, and selection, includes all biological fields and spreads to other areas such as philosophy. Medicine is an example of how the evolutionary perspective can greatly improve the understanding of concepts in an area, as human health and pathological conditions are under the effect of evolution. Evolutionary medicine is an emerging paradigm for understanding human heterogeneity, health, and diseases. Nevertheless, there are indications that medical research and practice are only marginally affected by these ideas. Here, we investigate how concepts of biological evolution are employed in medical research. We use a bibliometric approach to look for the presence and frequency of biological evolution-related concepts in medical articles. The distribution of these concepts over the years is analyzed according to the medical specialty and the impact of the journal. Our data showed that: i) only a small percentage of articles in medical journals have an evolutionary perspective; ii) medical journals where these evolution-based articles are published focus on basic science, theoretical medicine, and less frequently, on applied medicine; iii) these articles are mostly from the microbiology, immunology, neurology, psychology, behavior, and oncology fields; and iv) viruses are the most frequently covered microorganisms, followed by bacteria, fungi, and protozoans. The collection of our results, considering the importance of evolutionary medicine in the medical field, highlights the need for a decisive change in perspective in medical research.

## Introduction

Evolution is one of the most important concepts in biology. In 1859, Charles Darwin presented his theory of evolution in the book “On the Origin of Species” (1,2), in which he defined evolution as the result of “natural selection”, in which there is a struggle for survival among individuals of a biological species, and the more successful individuals tend to propagate the traits that contributed to their success. Evolutionary biologist Theodosius Dobzhansky said, “nothing in biology makes sense except in the light of evolution” (3). Accordingly, this concept should also be applied to medicine. All organs, tissues, cells, organelles, and molecules of a human being are subject to the forces of evolution, as is the whole individual. Therefore, all normal and pathological human conditions can be analyzed under the light of biological evolution. Many attempts have been made to understand how specific diseases have appeared and evolved in humans, and perhaps the most studied of all is cancer (4-6). Studying human pathological conditions from the perspective of evolution can help to understand the disease and its mechanisms and contribute to the development of effective treatments (7,8). Unless an evolutionary perspective is adopted, multiple pathologies run the risk of being poorly understood (7). Furthermore, as stated by Lozano (9), evolutionary thinking in medicine overlaps with the field of epidemiology, where long-term planning and solutions are needed.

Evolutionary medicine ideas began in the 19th century, and many new thoughts emerged during the 20th century (10-17), which strengthened this field. Evolutionary medicine can be defined as the intersection between evolutionary biology and medicine (18). This area started with the informal gathering of interest groups and evolved to the establishment of a scientific society and journal (19). An interesting example of evolutionary medicine can be found in clinical microbiology, where the integration of evolutionary biology, microbiology, bioinformatics, and clinical expertise is improving our understanding of how microbes influence human health (20). However, in a preliminary survey, we observed that most medical studies ignore evolution. Therefore, to evaluate how medicine deals with the concept of biological evolution, we applied a bibliometric approach to analyze these concepts within articles published in medical journals. Our data showed that only a minority of these articles employ an evolutionary perspective. We believe that this omission hampers the appearance and development of new ideas in the treatment of human diseases. We suggest that the inclusion of evolutionary concepts in the medical curriculum could benefit the formation of physicians with skills to understand how evolution shapes human disease.

## Material and Methods

### Construction of “Top 50 EvoMed Database”

The platform Scimago Journal Rankings (https://www.scimagojr.com/journalrank.php?category=2701&year=2020) was initially used to identify the top 50 ranked (by number of citations per article) scientific journals in the medical field. The parameters for this search were: medicine (miscellaneous), all subject areas, all types, all regions/countries, and the year 2020. The next step was to individually search each selected journal for articles associating biological evolution to medical fields. The raw Top 50 EvoMed database comprises all articles from each of the first 50 journals. However, the search was refined by excluding articles that use the concept of “evolution” in a meaning other than “biological evolution”. The textual analysis of each article of the raw Top 50 EvoMed database began by locating the word “evolution” in the text and selecting the corresponding sentence in which it appears. These were used as “classificatory sentences” of the articles. The resulting database was named “Top 50 EvoMed database” (21) and was subjected to further analysis.

In December 2021, the PubMed database (https://pubmed.ncbi.nlm.nih.gov/) was used for further search in each of the previously selected top 50 journals. In order to exclude non-biological meanings of evolution, the following syntax was applied for the advanced search settings, always replacing “xxxxxx” with the name of the journal being investigated: ((((((((((((((((((((((((((((((((((((((((((((((((((((((“evolution”[Text Word]) OR (“evolutionary”[Text Word])) AND (“xxxxx”[Journal]) NOT (evolution of the knowledge[Text Word])) NOT (evolution of the patient[Text Word])) NOT (evolution of the health)) NOT (evolution of the public)) NOT (evolution of the knowledge)) NOT (evolution of the term)) NOT (clinical evolution)) NOT (evolution in the treatment)) NOT (patient evolution[Text Word])) NOT (health evolution)) NOT (knowledge evolution[Text Word]) NOT (x-ray evolution[Text Word])) NOT (technical evolution)) NOT (evolution of the technique [Text Word])) NOT (evolution of donation)) NOT (case evolution)) NOT (evolution of the case[Text Word])) NOT (surface evolution)) NOT (contour evolution)) NOT (pathological evolution)) NOT (pseudo-wavelet evolution) NOT (graft evolution)) NOT (marching evolution)) NOT (evolution in perception)) NOT (transmural evolution)) NOT (evolution of the transmural)) NOT (angle evolution)) NOT (evolution of human cirrhosis)) NOT volution of blood-counting techniques)) NOT (evolution of in vitro diagnostics)) NOT (evolution of diagnose)) NOT (diagnose evolution)) NOT (evolution of inguinal)) NOT (evolution of the stress concept)) NOT (evolution of legal)) NOT (chronic evolution)) NOT (evolution of concepts)) NOT (evolution of hyperleukocytosis)) NOT (evolution of the vector cardiographic)) NOT (evolution of hypertensive)) NOT (evolution of pedagogic)) NOT (evolution and prognosis)) NOT (evolution of the medical)) NOT (evolution of our ideas)) NOT (evolution of military)) NOT (surgery evolution)) NOT (evolution of surgery)) NOT (evolution of cataract)) NOT (cataract evolution)) NOT (evolution in group practice)) NOT (evolution of medical)) NOT (endoscopic evolution)) NOT (evolution of clinical)) NOT (serologic evolution)) NOT (evolution of the surgical).

### Construction of the “Broad EvoMed Database”

To expand our search, a new database was designed for bibliometric evaluation of a more representative sample of the broad medical universe. Instead of focusing only on prominent journals, the goal here was to extend the search to include all journals with “Medical” or “Clinic” in the title, independent of impact ranking. Articles from the PubMed database were collected in October 2021 using the same textual analysis approach to exclude articles with other meanings for “evolution” (21). This syntax collects the words “evolution” and “evolutionary” in the text of just scientific journals containing “Med” and “Clinic” in the title, and the resulting articles were assembled to generate the raw “Broad EvoMed Database” (21).

In our manuscript, we aimed to analyze the use of biological evolution concepts in medical journals rather than publications focused on the field of evolutionary medicine. This is a subtle but important distinction. In the Top 50 EvoMed database, we aimed to analyze the use of evolution in medicine in the universe of highly cited journals and in the Broad EvoMed database we aimed to analyze a broader range of medical and clinical journals that publish papers related to evolution in medicine. Importantly, we did not simply use the entire PubMed database, because a PubMed search using the terms “evolut” and “medic” generates a total of 170,515 articles (online search as of July 13, 2023), most of which are not related to the use of biological evolution in medicine. A careful manual analysis would be difficult to do with such a large number of articles.

### Textual analysis

We analyzed the frequency of words in the title and in the classificatory sentence of the text of the selected articles. We chose the title and the classificatory sentence with the expectation that they would provide a concise representation of the whole article. Wordle software (created by Viégas et al. (22) and freely available at http://www.wordle.net) was used to generate a list of words with their relative frequencies and to generate “word clouds”. The clouds highlight words that appear more frequently in the source text, i.e., more frequent words appear with larger letters and in a colored gradient. The following parameters were used to generate the word clouds: removal of common English words and numbers, lower case all words, Telephoto font type, rounded edges, kindled color, and horizontal layout. Only words that appeared at least three times in the entire set of titles and classificatory sentences were used. The list of words was manually edited to remove plural words, different spellings of some words, and symbols. We also used the freely available software VOSviewer (https://www.vosviewer.com/) to analyze the frequency of words that appear in the titles and abstracts of the articles (23). A term map of co-occurrence relations between scientific terms was created (24) using the following parameters: all keywords, complete count, minimum number of occurrences of a keyword of 3, maximum length of circles of 100 words, and maximum size of lines of 1000 words.

## Results

### Word frequency in titles of articles with evolutionary concepts

**Figure 1:**
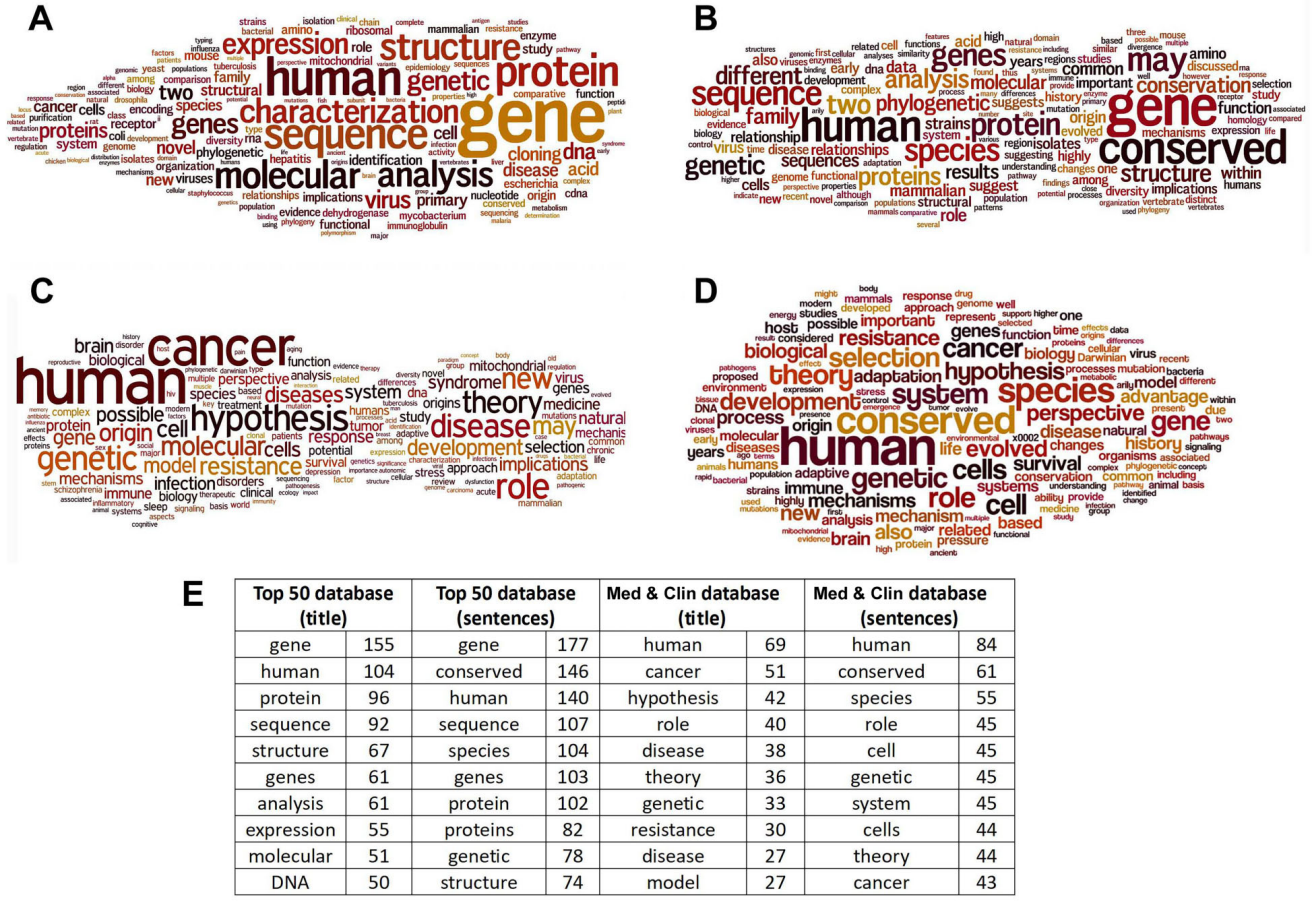
Vocabulary frequency of medical articles containing an evolutionary context. Word frequency in titles (**A** and **C**) and classificatory sentences (**B** and **D**) from the Top 50 (**A** and **B**) and Broad EvoMed (**C** and **D**) databases using word clouds. **E**, The ten most common words (and their frequencies) of each database.

First, the top 50 journals in the medical field were selected using Scimago. From these, we selected all articles that contained the words “evolution” or “evolutionary” using PubMed. A total of 2219 articles were selected from the Top 50 journals, and 572 articles were excluded after a manual text analysis, because they did not contain biological evolution concepts but other uses and meanings of the word “evolution”. Thus, sentences with a clear use of biological evolution concepts (“classificatory sentences'') were selected from the remaining 1647 articles. From the “Top 50 Evomed database”, word frequency in titles and classificatory sentences was determined using Wordle software (Figure 1A and B). Words representing basic human and medical science concepts, such as “gene”, “human” and “protein” were frequently found.

The medical literature that was evaluated was restricted to the Top 50 journals. This database may not represent the entire universe of medical journals. For this purpose, a new search was performed in PubMed for biological evolution in journals containing “Medical” or “Clinical” as the first word in the journal title, and 2746 articles were sorted out. Based on text analysis, 1761 articles were discarded and 985 articles from 111 medical journals were selected that associated medicine to biological evolution. Classificatory sentences containing this association were selected for each article and included as a new row in the broad database. This broad evolutionary-medicine database (“Med & Clin database”) was studied in the same way as the Top 50 database. Figure 1C and D shows the resulting word frequency (excluding “evolution”) in titles and classificatory sentences, respectively. Words representing basic human and medical science concepts, such as “human”, “cancer”, “disease”, and “role” were prominent in the clouds. Additional analysis revealed other recurrent concepts related to biological evolution and basic human and medical sciences. The table in Figure 1E displays the ten most common words (and their frequencies) in each database.

### Frequency of evolutionary concepts in medical journals

To determine the percentage of articles in the medical field with data related to biological evolution, we calculated the total number of articles published in journals containing “Medic” and/or “Clinic” in the title. A filtered PubMed search yielded 847,541 results from 1880-2021. Thus, the proportion of medical/clinical articles with an evolutionary perspective (986 articles) is about 0.116%, which clearly shows that only a minority of articles in medical journals meet this criterion. This percentage increased to 0.13% when we considered only the period from 2000 to 2021, when the overall global scientific production increased.

### Timeline of publications with evolutionary concepts

**Figure 2:**
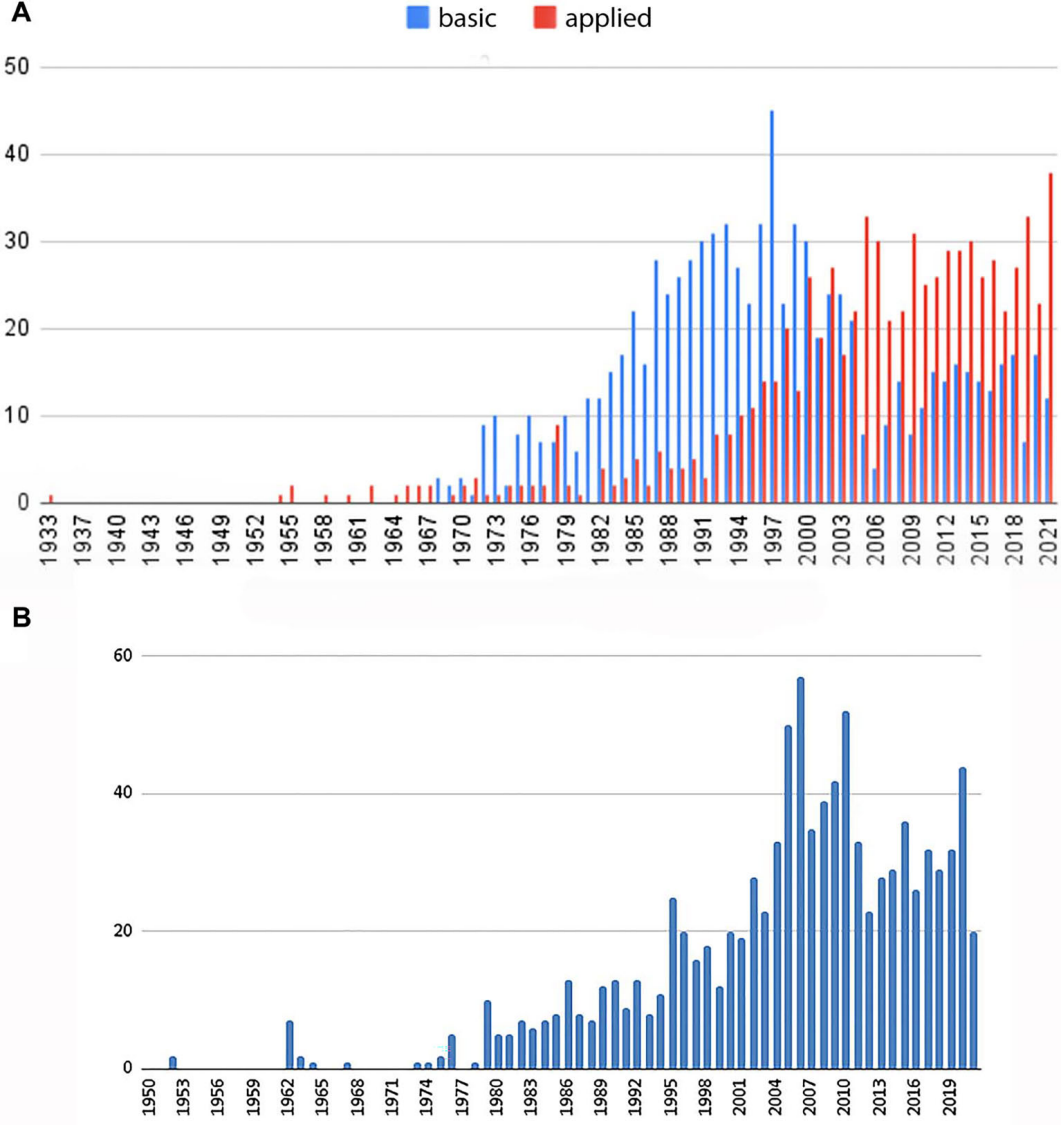
Distribution of medical articles containing an evolutionary context over the years. Blue bars represent journals of basic science and red bars represent journals of applied medicine. The Top 50 database is represented in panel **A** and the Broad EvoMed database is represented in panel **B**.

A chronological analysis of the Top 50 EvoMed database was performed to address how concepts in evolutionary medicine were used by authors over time. The first questions we examined were “When did the concept of evolutionary medicine first appear in articles?” and “What is the time course of the number of publications using evolutionary concepts?”. The Top 50 EvoMed database was intentionally extended to include other journals, such as the “European Journal” collection. These journals were not included in the top 50 positions of the Scimago Institutions Rankings, but we included them because they are prestigious journals that publish basic biomedical research. Among the 141 journals in the database, 20 journals were considered as basic medical science (Supplementary Table S1). Differences in the use of biological evolution concepts in articles over time in basic and more applied medical journals are also shown in Figure 2A. Basic science journals began publishing articles with evolution concepts in 1968 and reached the highest number of publications in the 1990s. In comparison, applied medicine journals began publishing articles with evolution concepts in 1933, and the peak number was reached only after 2000, a 10-year delay compared with basic science journals. Thus, by the end of the 1990s, the two groups of journals (basic and applied sciences) had opposite trends, with applied science journals showing a continuous increase in publications initiated in the 1990s, while basic science journals showed a decline after 2000.

Articles from the journals of the “Broad EvoMed Database” were assembled according to publication year, as shown in Figure 2B. Although these publications covered the period from 1951 to 2021, there is a clear increase in publications after 2000. Interestingly, this is the same profile of peak publication (after 2000) as observed in the Top 50 applied science journals. The journal Medical Hypotheses is almost 100 times more represented than most journals of the Broad EvoMed database. This overrepresentation significantly skews the distribution of Evo-Med correlation over time in the Broad EvoMed database.

### Comparison of use of medical concepts in articles from different medical fields

**Figure 3:**
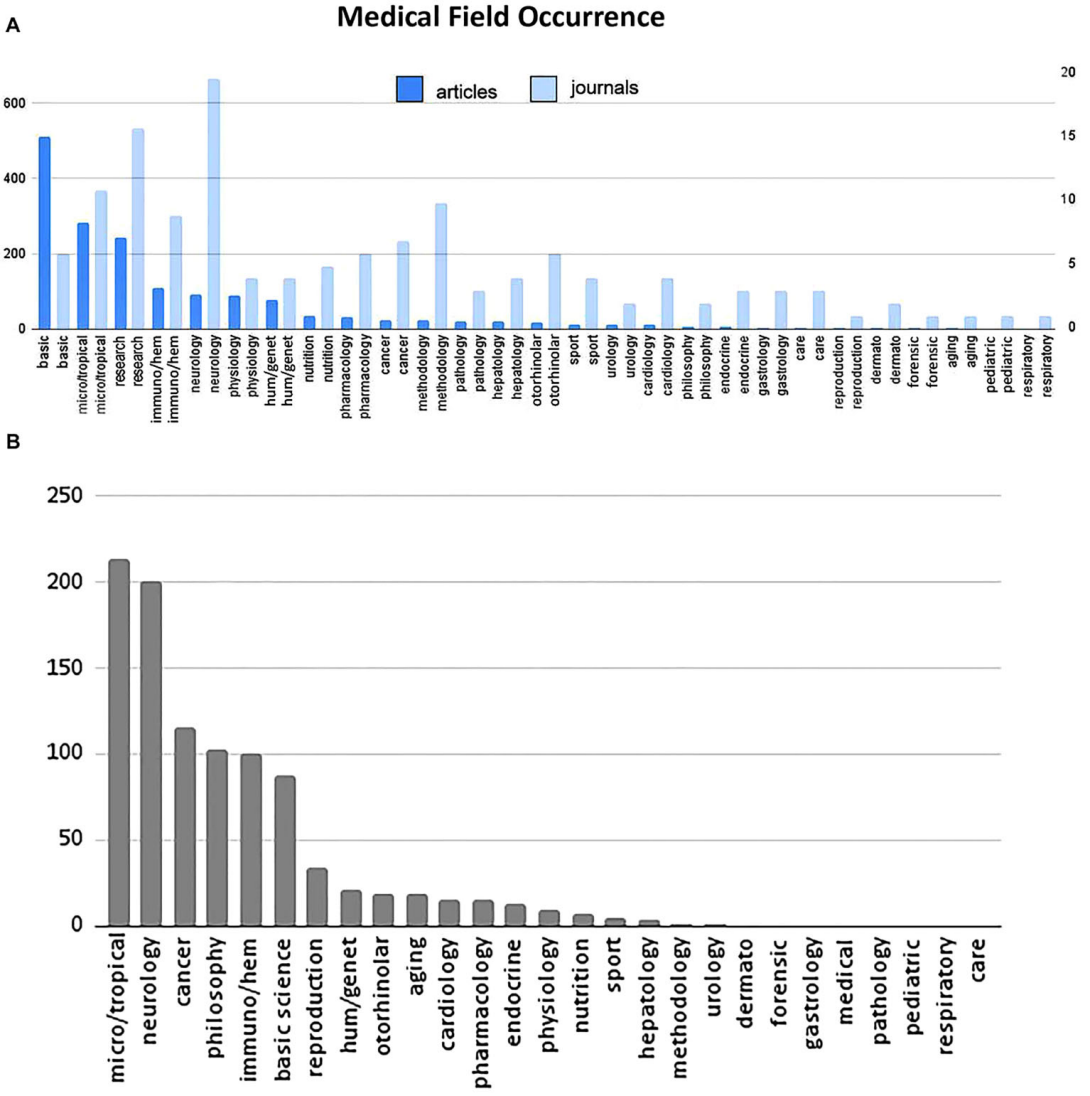
**A**, Frequency of medical articles (dark blue) and medical journals (light blue) with an evolutionary approach according to medical field. The Top 50 database is represented in panel **A** and the Broad EvoMed database is represented in panel **B**.

The medical field/specialty was revealed as pivotal for the use of biological evolution concepts. Based on journal titles, the journals were gathered according to medical field and the total number of articles for each field was counted (Figure 3A and Table 1). As expected, the fields with the most publications in biological evolution were: 1st) basic fields such as anatomy, biophysics, biochemistry, and cell biology (consisting of American Journal of Anatomy, European Biophysics Journal, European Cell Mater, European Journal of Biochemistry, European Journal of Cell Biology, and European Journal of Morphology); 2nd) microbiology, tropical science, and epidemiology; 3rd) journals with a broad scope (including JAMA, Lancet, New England Journal of Medicine, Nature Medicine, PLoS Medicine, and Science Translational Medicine); and 4th) immunology, hematology, physiology, neurology, psychology, and human genetics. Fewer than 34 articles were found in all other fields of clinical medicine. The number of journals for each field did not correlate strictly with the number of articles in that field (Figure 3A and Table 1). Neurology journals were the most represented in the database, despite having fewer articles. In contrast, journals with a broader scope were the second most represented in the database. Basic medical science was represented by only 6 journals, but was the most represented journal group in terms of the use of evolutionary medicine.

**Table 1:** Number of articles related to biological evolution published by each medical journal.

Journal	Number of articles
Eur J Biochem	425
Annu Rev Microbiol	112
J Clin Microbiol	106
Eur J Immunol	82
Eur J Hum Genet	62
Eur J Protistol	56
Am J Trop Med Hyg	55
Am J Physiol	52
Am J Anat	13
Eur J Cell Biol	37
Physiol Rev	35
JAMA	31
Eur J Neurosci	29
Eur Biophys J	24
Nat Med	23
N Engl J Med	21
J Clin Virol	20
Am J Clin Nutr	18
Am J Pathol	17
Cold Spring Harb Perspect Med	16
Eur J Pharmacol	15
Hepatology	15
Am J Psychiatry	14
Eur J Clin Microbiol Infect Dis	13
Lancet Infect Dis	13
Med J Aust	12
Eur J Immunogenet	11
Eur J Morphol	11
J Am Soc Nephrol	11
Ann Intern Med	10
Brain	10
Eur J Oral Sci	9
Am J Med	7
Eur J Med Chem	7
Lancet Neurol	7
Mol Ther Nucleic Acids	7
Am J Psychol	6
Ann Oncol	6
Annu Rev Nutr	6
Eur J Histochem	6
Eur J Nutr	6
J Clin Med	6
Am J Anat	5
Am J Cardiol	5
Eur J Cancer	5
Eur J Epidemiol	5
Eur J Med Genet	5
J Thorac Oncol	5
Sci Transl Med	5

Previously, the medical fields of the articles in the Top 50 database were determined based on journal titles. However, many journal titles in the Broad EvoMed database did not clearly inform the medical field classification. As a different approach, text analysis was tested to classify articles of the Broad database. To facilitate a comparison with the Top 50 database, the same field categories were used here to classify the articles by text analysis. As shown in Figure 3B, microbiology was the medical field with highest occurrence of evolutionary concepts, as expected. Nevertheless, besides microbiology, other fields such as neurology, oncology, physiology, immunology, hematology, and basic science were also revealed to be more frequent medical fields with a medical-evolution correlation. Interestingly, neurology, oncology, and physiology were not represented in the Top 50 EvoMed database.

### Relationship between concepts in selected evolutionary articles

**Figure 4:**
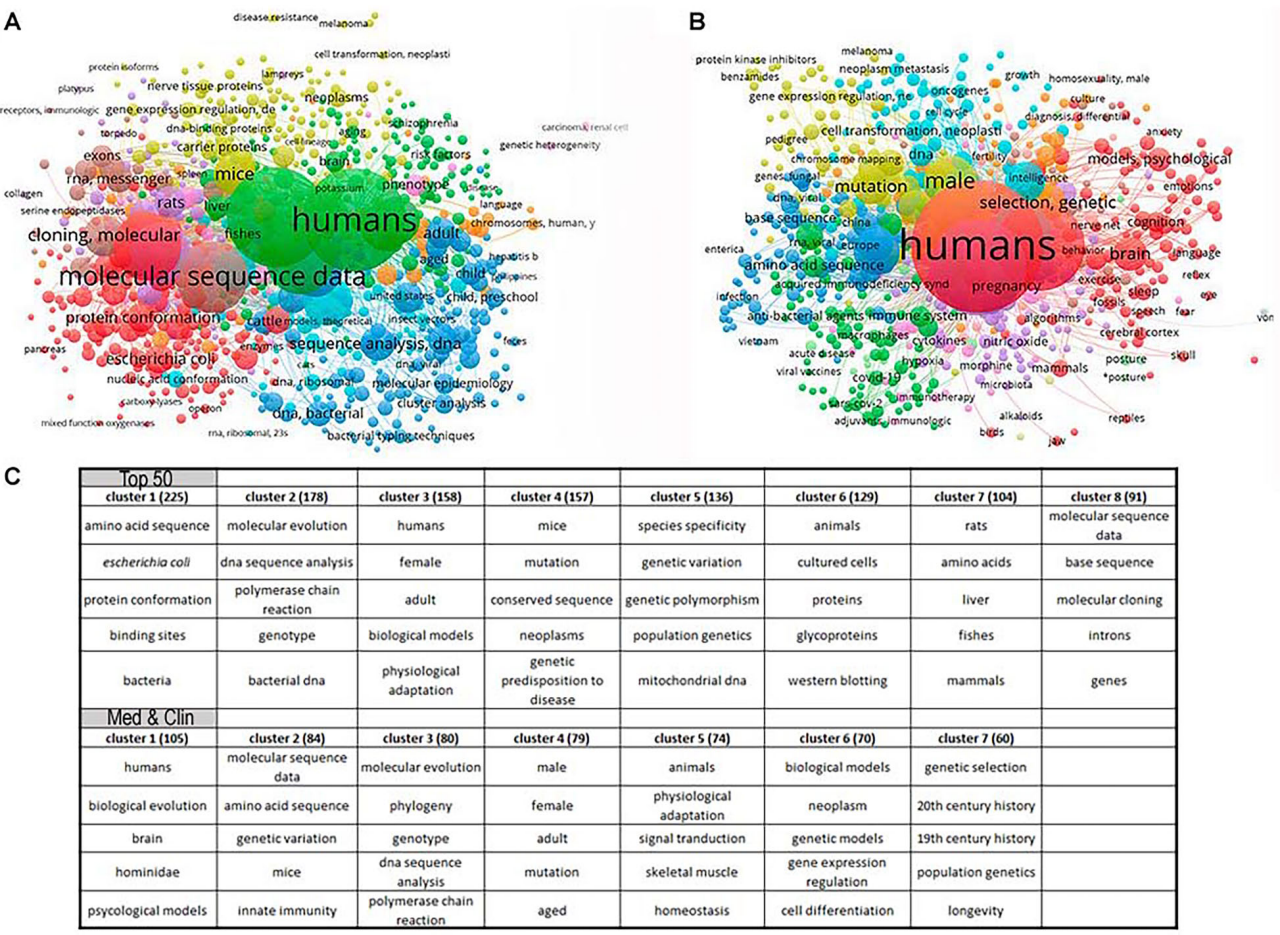
VOSviewer diagram showing the proportional number of concept occurrences and their relationship in medical articles with an evolutionary context. The size of each concept (circle) represents the number of occurrences. The lines, proximity, and colors indicate the relationships among concepts. **A**, Diagram related to the Top 50 database and **B**, diagram related to Broad EvoMed database. **C**, The five most frequent concepts related to each cluster in both databases (Top 50 and Broad EvoMed) obtained with VOSviewer software.

The connection of the subjects associating medical fields with biological evolution was addressed using VOSviewer software. Article titles of the Top 50 database were used to generate concept connective maps (Figure 4A). The program was set to consider concepts occurring at least 4 times, since it generates the maximum possible number of concepts for the output map. The size of the concept keyword in the map and the distance among the concepts correlates with the number of occurrences and the strength of the relationship among them, respectively. The map shows amino acid sequence, molecular sequence data, animals, humans, and biological evolution as central and linked to most concepts (Figure 4A). The software generated colors to identify groups of interlinked relationships (clusters), as shown in the map. A proposed classification for these groups of interlinked concepts is: i) “molecular biology cluster” in brown, with cloning, sequence, and genes; ii) “experimental model cluster”, in purple, with chicken, rat, and DNA sequence; iii) “microorganism cluster” in red, with protein sequence, *E. coli*, Saccharomyces, enzyme, bacteria, and microorganisms; iv) “medical cluster” in blue, with human age, virus, bacteria, sequence, epidemiology, and molecular evolution; v) “human cluster” in green, with human gender, animals, biological evolution, and neurology/behavior; and vi) “mice cluster” in yellow, with mice, cell biology, and mutation.

The connection of the subjects in the broader medical universe was also addressed by VOSviewer analysis, as described for the Top 50 EvoMed database. Reference titles and classificatory sentences of the Med & Clin database were thus processed to connect all words cited 3 or more times, and this analysis generated the concept's connective map for the broader medical universe (Figure 4B). VOSviewer generates the maximum possible number of concepts in the output map. The area of the concept on the map and the distance among concepts indicate the number of occurrences and the strength of the relationship, respectively. The map shows “humans”, “animals”, “biological evolution”, and “biological models” as central concepts connected with most others, as expected. The colors of the concepts were used to identify connected groups, as shown on the map. The proposed classification for groups of interlinked concepts is: yellow for human gender, age groups, and ageing; green for virus, immune system, mice, and pregnancy; dark blue for phylogeny, molecular evolution, sequence analysis, genomics, molecular epidemiology, and microorganisms; purple for animals, signal transduction, physiology, stress, and inflammation; red for humans, biological evolution, nervous system/behavior, and reproduction; orange for genetic selection, history, population genetics, vectors, and mitochondrial DNA; and light blue for models, neoplasms, environment, life origin, cell differentiation, and cell cycle. The table in Figure 4C shows the five most frequent concepts related to each cluster obtained with VOSviewer software in both databases (Top 50 and Broad EvoMed).

## Discussion

We have applied a bibliometric approach to analyze the use of biological evolution concepts in medicine. The collection of our data on “evolution+medicine” articles shows that: i) only the minority of articles in medical journals (less than 2%) had an evolutionary perspective; ii) evolutionary concepts were more frequently used in basic science and theoretical medicine articles than in applied medicine articles; iii) medical journals where most of these articles were published could be classified into basic science, theoretical medicine, and applied medicine, but not into medical specialties such as urology, pediatrics, and cardiology; iv) the chronological analysis of publications in “evolution+medicine”, beginning in 1933, showed a change in evolutionary medicine concepts from basic to broad theoretical medicine journals (such as Medical Hypotheses), and a steady increase in the number of “evolution+medicine” articles in applied medicine journals since the 1990s; v) among medical specialties, microbiology, immunology, neurology, psychology, behavior, and oncology contained most of the articles with the subject “evolution+medicine”, and vi) among microorganisms, viruses were the most represented in articles related to “evolution+medicine”, followed by bacteria, fungi, and protozoans.

In the Top50 database, the most frequent words were “gene”, “human”, and “protein”. In the Broad EvoMed Database, the most frequent words were “human”, “cancer”, “disease” and “role”. The high frequency of the word “human” in both databases could be attributed to the medical universe that was analyzed. The higher frequency of the terms “gene” and “protein” in the Top 50 database may arise from the molecular and cellular perspective of these highly cited journals. While the high frequency of the word “cancer” in the Broad database could be due to the well-established relationship between cancer and evolution, we could not speculate why “cancer” was not particularly frequent in the Top 50 database.

The first articles containing both evolution and medicine were published in the 1970s, and the number of this type of article increased until 1990, when it became stable in both databases studied (Figure 2A and B). Using the broad database, it was possible to observe that basic science articles were more frequent in the beginning, while applied medicine articles became more common later (Figure 2B). The chronological analysis reinforced the notion of a flow of information in EvoMed from basic to broad medical review journals over time and the constant increase in applied medicine journals in both databases over time.

High-impact journals probably paved the way and legitimized this trend in applied science journals, since there is a 10-year delay between the growth curves of these two groups. However, the first four journals of the Scimago impact ranking (New England Journal of Medicine, Nature Medicine, Physiology Review, and Lancet) published very few articles with the evolution-medicine combination. Nevertheless, articles linking evolution to medicine were more frequent and began to appear first in journals of basic medical science and reviews than in specific medical fields. Medical fields with a significant number of evolution-related articles include microbiology, epidemiology, and immunology, which could be related to the fact that evolutionary changes occur relatively fast and can be experimentally observed in microorganisms (Figure 3).

In our study, we found that the journal Medical Hypotheses was almost 100 times more frequent than most journals in the Broad EvoMed database. This overrepresentation is most likely related to the main interest of this journal, which is theoretical papers related to medicine. Importantly, the profile of this journal was questioned by the scientific community in 2010 (https://sciencebasedmedicine.org/is-there-a-role-for-speculative-journals-like-medical-hypotheses-in-the-scientific-literature/).

Viruses are the microorganisms most commonly mentioned in medical studies that contain biological evolution concepts, which is particularly important in view of the recent SARS-CoV-2 virus outbreak. The percentage of articles on the evolution of viruses and virus infections in humans is expected to increase in the next years. Other disease-related organisms, such as protozoa, appeared with an extremely low percentage in medical articles that contained evolution. These data are disturbing since these unicellular organisms are responsible for several parasitic infectious diseases in humans, such as malaria, giardia, Chagas disease, leishmaniasis, and toxoplasmosis, affecting the health and lives of millions of people every year worldwide (25). The consequences of the interaction between humans and protozoa need to be understood from an evolutionary point of view to facilitate treatment and epidemiological control.

Other medical fields with frequent articles on evolution were neurology, psychology, behavior, and oncology. This revealed how evolutionary concepts are now framing current views of diseases of the nervous system and cancer. Studies on the evolution of vertebrate neurological systems are pivotal to the understanding of how human neurological disorders emerged and evolved and their correlation with population genetics and environmental factors. In relation to cancer, it is vital to study how and when tumor cells first appeared in the animal kingdom and how they evolved in different tissues, organs, and species. An elevated occurrence of cell heterogeneity (both genomic and transcriptomic) is characteristic of several tumor cell masses and it correlates with patient survival (26). Understanding the characteristics of tumor cell heterogeneity and its evolution based on Darwinian natural selection can improve the development of new tools for the treatment of cancer in human patients.

VOSviewer analysis of medical articles in both databases (Top 50 and Broad EvoMed) showed that “humans” was by far the most frequent word and was the central node of interaction with all other words, which could be explained by the human-related nature of medicine (Figure 4A and B). In the Top 50 database, “molecular sequence data”, “molecular cloning”, and “DNA sequence analysis” were the following most frequent words, suggesting that most highly cited articles related to evolutionary medicine contained a molecular approach (Figure 4A). “Genetic selection” and “mutation” were highly frequent words in the Broad EvoMed database, suggesting a genetic approach in these articles (Figure 4B). The diversity of concepts found in the words of both databases shows that medical articles containing an evolutionary view cover a wide range of research questions and areas of basic and applied medicine. Using VosViewer, we identified seven-word clusters, and suggested criteria for their organization.

The modest use of evolutionary concepts (less than 2%) in medical journals that we observed was constant over the years, with some exceptions, and was not related to any specific type of medical journal. Biological evolution is well accepted in the scientific community, but its use to interpret events and structures in medicine is not common. As the analysis of the broader database showed, only 0.1% of the total number of publications in this database associated medical issues with biological evolution. Even during the most active period of our survey (2000-2021), the proportion of medical articles mentioning evolution did not increase significantly (0.13%). This suggested that the increase in scientific publications over the years did not affect the use of evolutionary concepts in the medical field.

Alcock (27) showed that the publication of articles on evolutionary topics increased steadily from 1991 to 2010. A PubMed search using the MeSH terms Biological Evolution and Medicine identified only 5 publications in 1991, 209 in 2010, with a maximum of 277 publications in 2009. The increase in PubMed publications corresponded to an average annual rate of increase of 26.5%. In contrast, our results showed the number of medical articles containing biological evolution has increased only 0.13% in recent years. The difference in these results could be explained by the methodology used to obtain the articles in both works. We used Scimago Journal Rankings (SJR) and Alcock (27) used PubMed database. SJR indicator is a numerical value that indicates the average number of weighted citations in a given year per paper published in that journal during the previous three years, as indexed by Scopus. We used SJR to select the top 50 journals in the medical field, while Alcock (27) used PubMed to select articles that were published on biological evolution in medicine. Therefore, our universe was probably larger than that analyzed by Alcock (27).

The few publications on evolution/medicine contrasted with the emergence of the revolutionary technology of DNA manipulation and sequencing. Sequence analysis and gene function studies from basic research revealed how evolution framed our understanding of biological structures and processes. Molecular biology data fed medical analysis bringing evolutionary understanding into medical practice. Our data also showed a tendency for authors with a bioinformatics approach to be the most active in making evolutionary associations in the medical field, thus corroborating our interpretation of the information flow paradigm after the emergence of DNA recombinant technology.

In conclusion, we believe that the data described here point to an urgent need for a deeper understanding of the concepts of Darwin's theory of evolution by medical students worldwide. Knowledge about biological evolution provides physicians with an integrative framework that links otherwise disparate bits of knowledge (28). In agreement with this idea, it has been suggested that premedical students need evolution courses in their curriculum (28,29). This is especially relevant considering the current advances in the bench-to-bedside strategies and efforts to improve health care worldwide. This conceptual merging of the so-called “basic” medicine with clinical practice has resulted in the entry of many biologists on the academic staff of medical schools, and this has been driven largely by advances in molecular (personalized) medicine, as well as pathology (forensics) and especially epidemiology. Incorporating the consequences of evolutionary forces of into the medical field could provide new insights into the understanding of pathological conditions and the development of new strategies for the prevention and treatment of new and old diseases, which we believe is related to the emergence of a new research field called evolutionary medicine (30). Evolutionary medicine, or Darwinian medicine, uses the principles of evolutionary biology to better understand, prevent, and treat diseases (31).

## Supplementary Material

Click here to view [pdf].
